# Negative bone scintigraphy in wild-type transthyretin cardiac amyloidosis

**DOI:** 10.1186/s12872-020-01749-x

**Published:** 2020-10-29

**Authors:** Nicolò Martini, Stefania Rizzo, Cristiano Sarais, Alberto Cipriani

**Affiliations:** Department of Cardio-Thoraco-Vascular Sciences and Public Health, University of Padua, Via Giustiniani, 2, 35128 Padua, Italy

**Keywords:** Transthyretin, Cardiac amyloidosis, Bone scintigraphy, Cardiac magnetic resonance, Immunoelectron microscopy, Case report

## Abstract

**Background:**

Amyloidosis is a rare systemic disease due to the extracellular tissue deposition of a fibrillar-shaped misfolded protein, called amyloid. Only two types of proteins commonly affect the heart leading to an infiltrative cardiomyopathy: immunoglobulin light chain and transthyretin (TTR) cardiac amyloidosis (CA). Despite the promising role of emerging imaging modalities, such as strain echocardiography, cardiac magnetic resonance and bone scintigraphy, its diagnosis is still often missed or delayed due to their inherent limitations and to a nonspecific clinical scenario with frequent concomitance of cardiac comorbidities. The gold standard for a definite diagnosis still remains endomyocardial biopsy, but in rare cases Congo Red staining could provide false negative results, as in our case, requiring immunoelectron microscopy.

**Case presentation:**

A middle-aged male adult presented to the emergency department for relapse of heart failure. Echocardiography and cardiac magnetic resonance, along with the history of bilateral carpal tunnel syndrome, were suspicious for TTR-CA. The diagnosis, however, was hampered by concomitant cardiac comorbidities and conflicting results of imaging modalities. In fact bone scintigraphy was negative, as well as Congo Red Staining on myocardial tissue samples obtained by endomyocardial biopsy. Given the high clinical suspicion, immunoelectron microscopy was performed, showing TTR amyloid fibrils deposits, that confirmed the diagnosis. A genetic analysis excluded and hereditary form. The patient was then referred to a specialist center for specific treatment.

**Conclusions:**

This is a rare case of a TTR-CA with a negative Bone Scintigraphy and Congo red staining, which demonstrated that CA is frequently misdiagnosed because of the low specific clinical manifestations and the results of imaging modalities that sometimes could be misleading, with subsequent delayed diagnosis and correct treatment.

## Background

Amyloidosis is a rare systemic disease due to the extracellular tissue deposition of amyloid, which is a substance composed by insoluble misfolded proteins organized in a fibrillar manner [1]. Several endogenous proteins have been reported to show amyloidogenic behavior, but only two types commonly affect the heart leading to an infiltrative/restrictive cardiomyopathy: immunoglobulin light chain (AL) amyloidosis and transthyretin (TTR) amyloidosis, the latter divided into wild-type and hereditary forms according to the presence or absence of a genetic substrate. The diagnosis is challenging and is frequently missed or delayed, because of the low specificity of clinical manifestations [2]. Moreover, coronary artery disease (CAD) frequently occurs in patients with cardiac amyloidosis (CA), with the effect of drawing the attention of clinicians and further delaying the diagnosis.

A histological demonstration of heart infiltration and the identification of the precursor protein causing amyloid are generally recommended for a definite diagnosis. However, endomyocardial biopsy (EMB) is an invasive procedure, also limited by sampling error and lack of generalized expertise in its performance. In the last two decades, strain echocardiography, bone scintigraphy (BS) and cardiac magnetic resonance (CMR) have acquired an established role in the clinical pathway of CA, by allowing a noninvasive and non-histological diagnosis of most cases of TTR-CA [2]. However, the diagnostic accuracy is not 100%. Herein, we describe a case of a male adult, whose TTR-CA diagnosis was hampered by concomitant cardiac comorbidities and conflicting results of imaging modalities, in particular the negative results of the BS.

## Case presentation

**Fig. 1:**
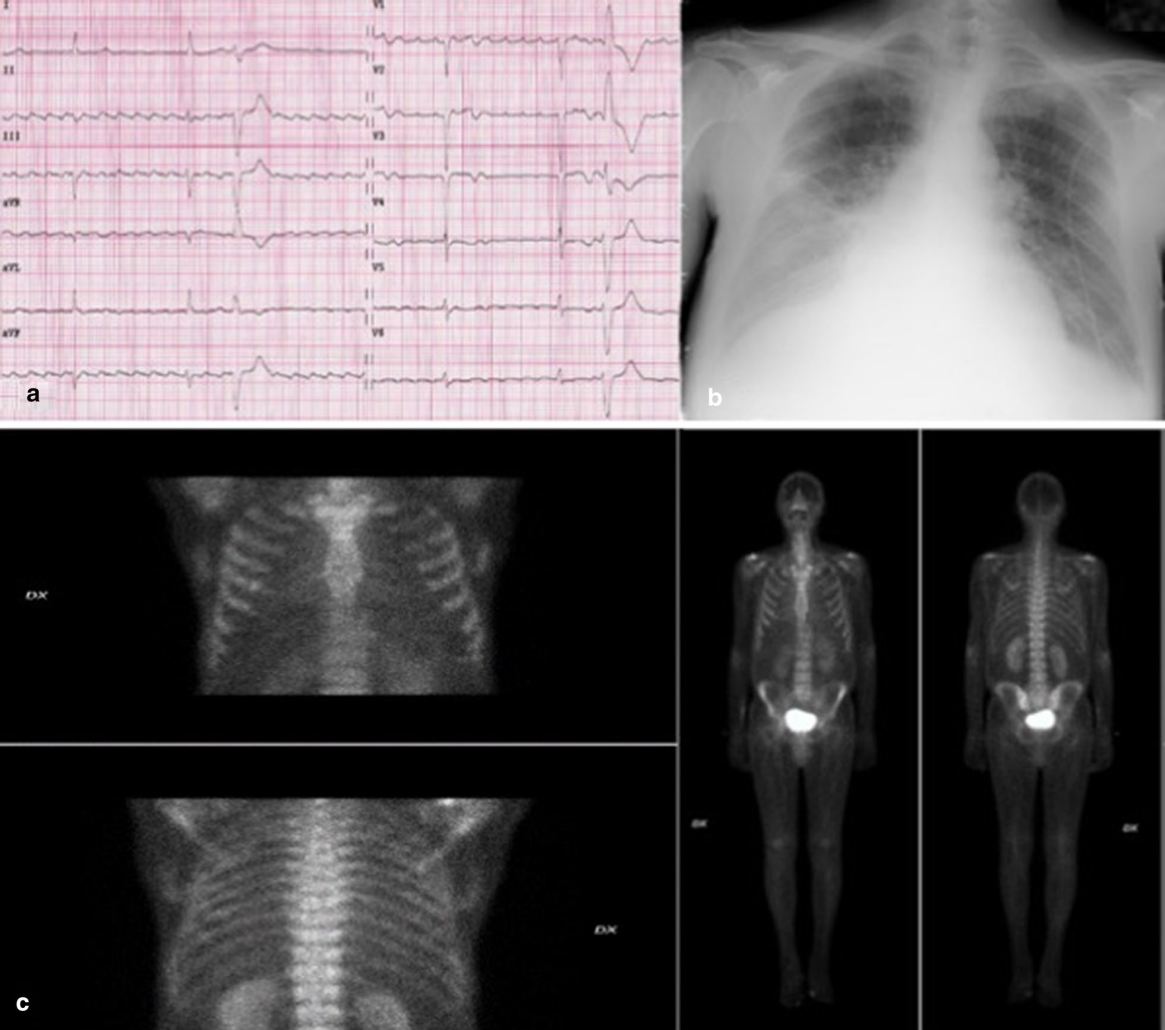
Electrocardiographic, chest X-ray and bone scintigraphy results. Electrocardiogram revealing atrial flutter with high degree atrio-ventricular block, left anterior hemiblock, pseudo-necrosis pattern, plus rare premature ventricular contractions (**a**). Chest X-ray showing hilar congestion and a severe pleural effusion (**b**). ^99m^Tc-DPD bone scintigraphy detecting no uptake of tracer in cardiac region (**c**)

**Fig. 2:**
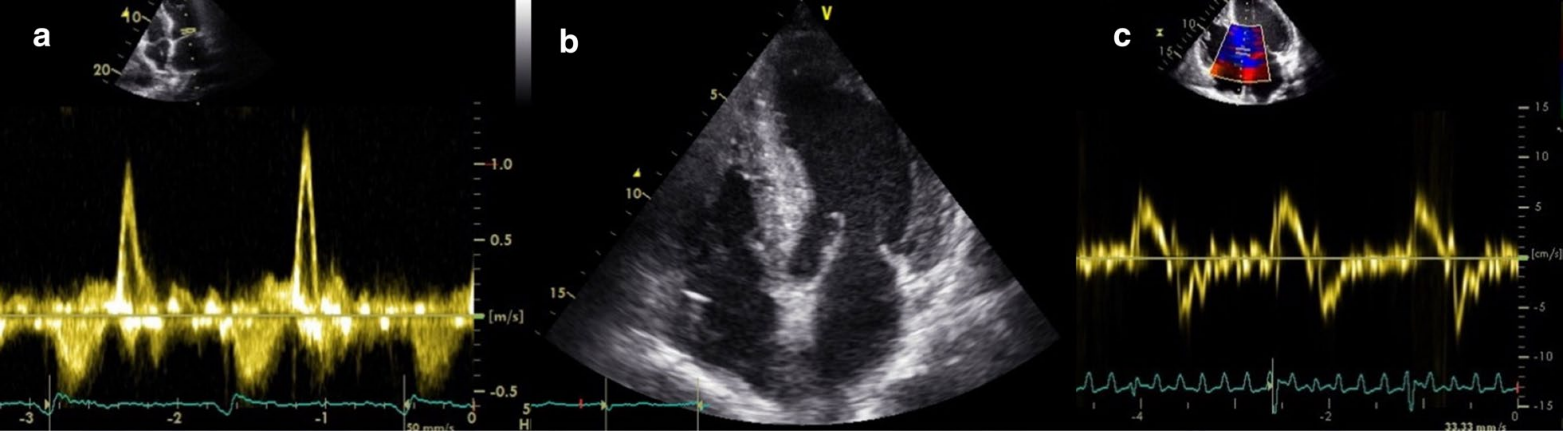
Echocardiographic results. Echocardiographic images showing a restrictive pattern of LV filling, characterized by a high E wave and reduced deceleration time on diastolic mitral valve inflow (**a**); biventricular hypertrophy and biatrial enlargement are appreciable in **b**; signs of high filling pressures, demonstrated by a reduced e′ velocity with high E/e′ ratio, are visible on **c**

**Fig. 3:**
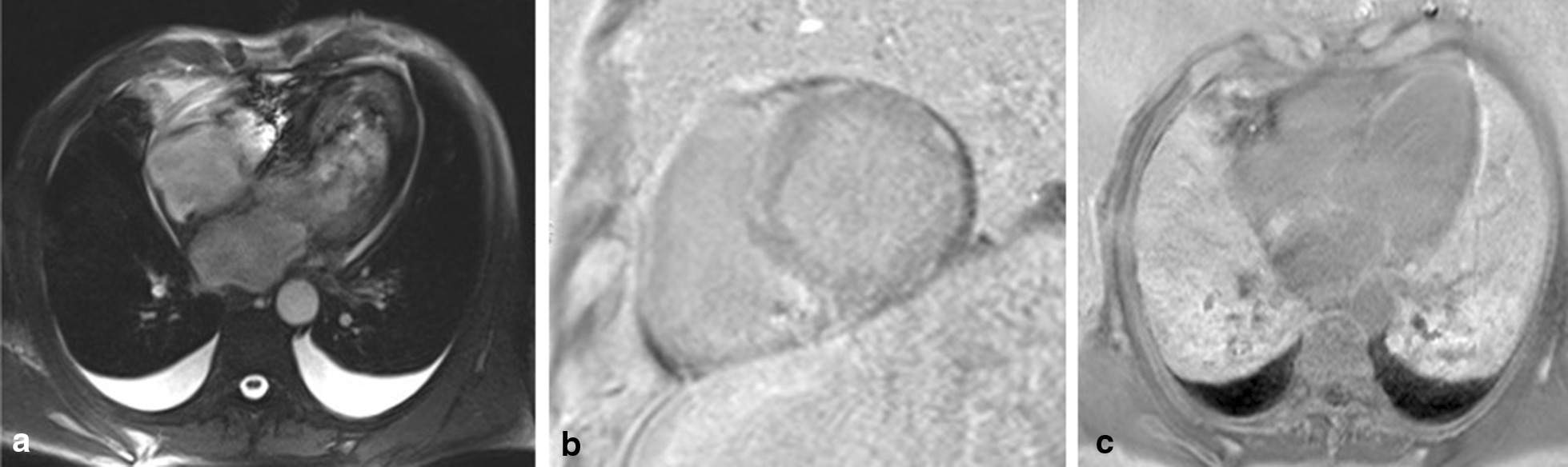
Cardiac magnetic resonance findings. Cardiac magnetic resonance images in cine long axis view (**a**) and post contrast long axis (**b**) and short axis view (**c**). Despite pacemaker artifacts, a hypertrophic left ventricle with diffuse myocardial late gadolinium enhancement is appreciable

**Fig. 4:**
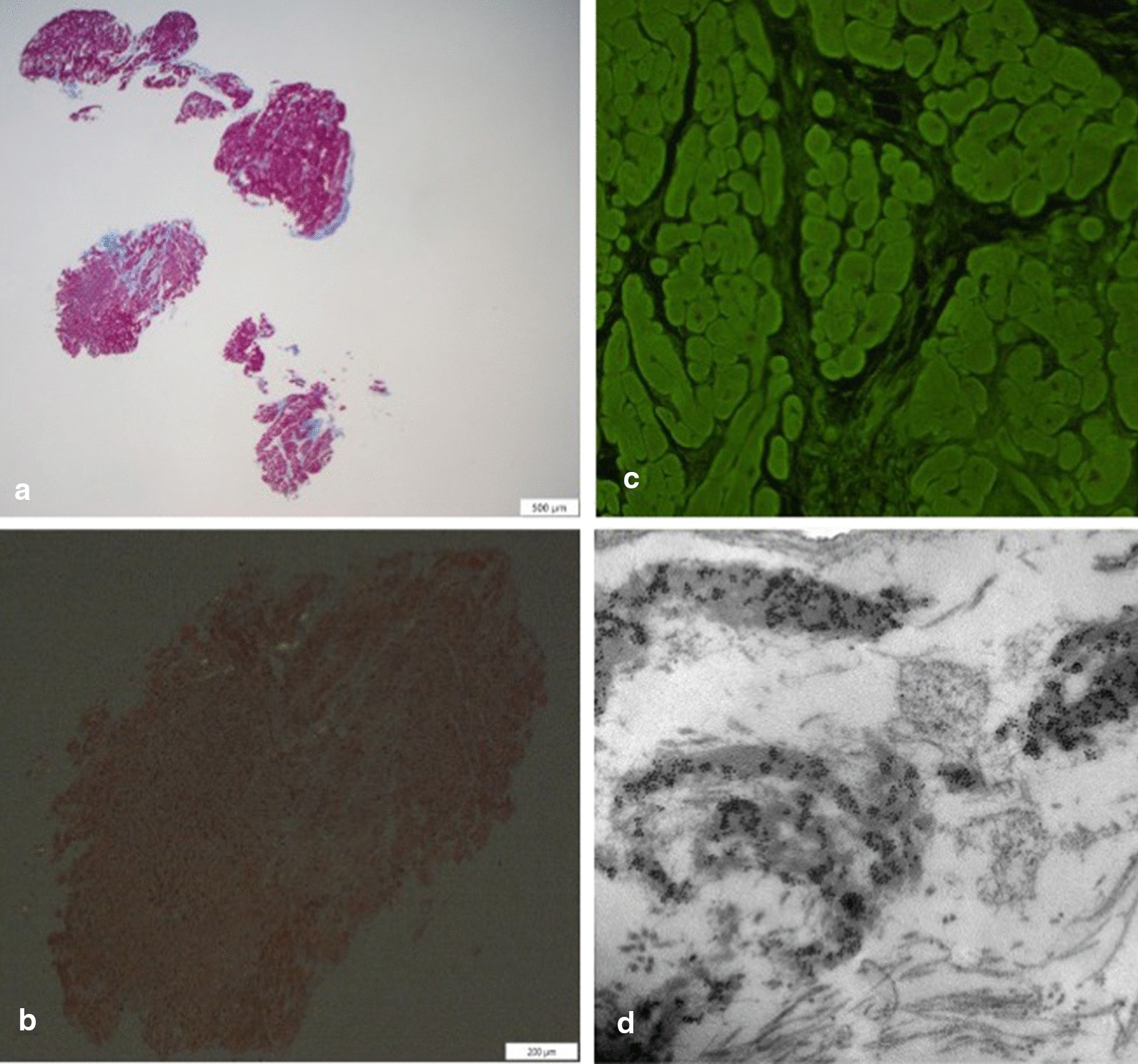
Endomyocardial biopsy findings. Panoramic view showing replacement type fibrosis (Azan-Mallory, scale bar 500 micron) (**a**). Congo Red staining was negative without the classical apple-green birefringence under polarized light microscope (Congo Red, scale bar 200 micron) (**b**). Thioflavin T fluorescence assay was negative (thioflavin T, original magnification ×20) (**c**). At immunoelectron microscopy, TTR amyloid fibrils are evidenced in the interstitial space (immunoelectron microscopy, scale bar 500 nm) (**d**)

The 67-year-old man who last January 2020 presented to the Emergency Department of our hospital for relapse of heart failure (HF) was already known to our Cardiology Unit. His medical history had begun in 2015 with an acute anterior myocardial infarction, treated with percutaneous angioplasty and stenting on left anterior descending coronary artery. That time, a CMR had been performed before discharge, showing a mildly dilated left ventricle (LV), slight decrease of LV systolic function (LV ejection fraction [EF] 47%) and a subendocardial late gadolinium enhancement (LGE) involving the apical and septal segments. Five years later, because of a new-onset atrial flutter and a severe pleural effusion (Fig. 1a, b), he had been admitted again to our hospital. Echocardiogram had revealed the signs of the previous apical myocardial infarction, and besides, a biventricular hypertrophy (LV mass 116 g/m^2^, interventricular septal and posterior wall thickness 1.1 cm and 1.4 cm, respectively), LV high filling pressures (E/E′ 27) and a severe biatrial enlargement (left and right atrial volume 56 cm^3^ and 51 cm^3^, respectively) (Fig. 2). Pleural effusion had been treated with thoracentesis. Because of the telemetry detection of high-degree atrio-ventricular block with long pauses (> 6 s), a single-chamber pacemaker had been implanted. Six months after the discharge, he came again for fatigue and dyspnea. Laboratory tests showed high cardiac troponin and high B-type natriuretic peptide, and the recurrence of severe pleural effusion required a thoracic drainage. Given the rise of troponin, and the echocardiographic worsening of LV EF, coronary angiography was repeated, showing a critical three-vessel disease. For testing myocardial viability and proceeding to coronary revascularization, CMR was performed and, despite image artifacts due to pacemaker, showed a hypertrophic LV with reduced systolic function (LV mass 97 g/m^2^, LV EF 35%) due to diffuse akinesia (Fig. 3a; Additional file 1: Video 1). Pleura and pericardium effusion were well appreciable. Inversion time scout sequence was not able to identify the appropriate inversion time to null normal myocardium (Additional file 2: Video 2), suggesting an abnormal gadolinium kinetics. Post contrast images detected a diffuse transmural LGE (Fig. 3b, c), a pattern which raised the suspicion of CA. However, a subsequent BS with technetium-99 m-3,3-diphosphono-1,2 propanodicarboxylic acid (^99m^Tc-DPD) did not detect any cardiac uptake of tracer (Fig. 1c). AL amyloidosis was ruled out by urine and serum immunofixation. A careful revision of the CMR findings, combined with the recent medical history, forced us to perform EMB, which at first sight showed replacement-type fibrosis and no evidence of amyloid deposits at Congo Red staining under polarized light microscope as well as at thioflavin T fluorescence [3]. Despite this, given the high clinical suspicion of CA, immunoelectron microscopy against kappa and lambda light chains and TTR was performed, showing some TTR amyloid fibrils deposits with diameters of about 9 nm in the extracellular matrix of the myocardium (Fig. 4). A genetic testing was then performed, but no pathogenic variants were detected, so that a wild type TTR-CA diagnosis was made. The patient was then discharged with anticoagulant, aspirin, statin, diuretic and mineralocorticoid receptor antagonist therapy, and referred to a tertiary care center for evaluation of TTR-targeted treatment. At 6-months follow up, he did not experience readmissions to hospital for HF.

## Discussion and conclusion

The diagnosis of CA is known to be complicated, for many different reasons.

First, clinical scenario is non-specific, including symptoms and signs like exertional dyspnea, palpitations, fatigue, weakness, fluid retention, which typically characterize the HF syndrome. Identifying the etiology of HF can be time and resources consuming and is usually limited to coronary artery or cardiac valves evaluation, because of the prompt availability of effective treatments like coronary angioplasty or valve replacement. The further management of HF generally is aimed to relieve symptoms, to maintain fluid balance and to control arrhythmias, and sometimes it can be done irrespective of the specific etiology. The clinical course of our patient well represents this strategy: coronary angiographies, pacemaker implantation and multiple thoracic drainages were required to control symptoms and improve outcome, but all of these procedures did not lead to a long-term benefit.

Second, it is not uncommon that once the ischemic etiology is established, all other causes of HF are sidelined. HF etiologies are roughly categorized into two main groups: ischemic and non-ischemic. Since the two categories have different management, therapy and outcome [4], they are often considered like two distinct entities, which rarely overlap. If a patient has been diagnosed with CAD, all medical efforts are consequently aimed at managing cardiovascular risk factors, reducing ischemia and improving or restoring the coronary blood flow. However, as well as CAD, also the prevalence of TTR-CA increases with age, leading these entities coexist in many patients and possibly influence each other. Indeed, CA may worsen a preexisting CAD, because of the higher LV mass and workload, lower capillary density, coronary extraluminal compression or intraluminal accumulation [5–7]. Importantly, identifying CA in patients with CAD is critical for therapy management: beta blockers and antihypertensive drugs are first line agents among CAD patients, while they are generally poorly tolerated in those with CA [4]. In our patient, the diagnosis of CA was “obscured” by the first diagnosis of CAD, and delayed despite the presence of CA-suggesting symptoms like fatigue and dyspnea, and signs like biventricular hypertrophy, altered cardiac biomarkers, high-grade AV block and recurrent pleura effusion.

Third, the old age of patients commonly discourages the clinicians to perform advanced diagnostic procedures. Furthermore, cardiac imaging modalities are not free of limitations. The hallmark of CA on echocardiogram, such as increased left ventricular thickness, atrial enlargement, and preserved or reduced LV systolic function, can be present in other conditions with increased afterload, such as aortic stenosis, hypertrophic cardiomyopathy or hypertensive heart disease [8]. Likewise, the severe impairment of diastolic function, characterized by a restrictive pattern of LV filling (high E wave and low A-wave velocity, increased E/A ratio, and reduced deceleration time on diastolic mitral valve inflow), and high filling pressures on tissue Doppler imaging (mitral and tricuspid annular e′ velocities markedly reduced, with a high E/e′ ratio) is not specific for CA.

An apical sparing pattern at longitudinal strain echocardiography has been reported to have high diagnostic accuracy for CA among patients with increased LV wall thickness [9]. However, in our patient, strain echocardiography was not reliable because of concomitant CAD and the previous (apical) myocardial infarction. Bone scintigraphy with technetium-labeled bisphosphonates is a well-established non-invasive method to diagnose TTR-CA. Although its sensitivity was recently demonstrated to be low in patients with a specific TTR mutation [10], for all other TTR-CA including the wild-type, BS showed high sensitivity [11]. However, the case herein reported is a rare example of wild-type TTR-CA with a negative BS, which could be possibly due to the scant amount of amyloid infiltrating the myocardium, as observed by the EMB, possibly representing an early stage of the disease.

Tissue characterization by CMR and EMB were crucial to achieve the diagnosis of TTR-CA in our patient. In amyloidosis, the abnormal myocardium signal can be measured using T1-/T2 mapping, LGE, and extracellular volume (ECV) imaging. Native T1 and ECV are markedly increased in CA [8]. Furthermore, global subendocardial, diffuse or transmural LV LGE and difficult nulling of the myocardium are specific features of CA, although they do not allow to differentiate between TTR and AL. EMB and the subsequent histological and immunoelectron microscopy analysis gave us the opportunity to make the final diagnosis and to characterize the type of amyloid fibrils, fundamental for a correct treatment planning [12].

Our case report demonstrated that TTR-CA is a disease whose diagnosis is often missed or delayed due to its nonspecific clinical scenario and frequent association with other cardiac comorbidities, like CAD. Cardiac imaging modalities are promising, but carry inherent limitations that should not be overlooked. EMB with immunoelectron microscopy provides certain diagnosis.

## Supplementary information

**Additional file 1: Video 1**. CMR cine images. CMR cine images in long-axis view revealing a hypertrophic LV with reduced systolic function due to diffuse akinesia. Pleura and pericardium effusion are also appreciable.

**Additional file 2: Video 2**. CMR post contrast images. CMR inversion time scout sequence in mid short-axis view showing the abnormal kinetics of gadolinium. Note that the inverted relationship between blood and myocardium blood pool, because of amyloid infiltration.

## Data Availability

All data presented in this report are available from the corresponding author on reasonable request.

## Abbreviations

BS: Bone scintigraphy
CA: Cardiac amyloidosis
CAD: Coronary artery disease
CMR: Cardiac magnetic resonance
EF: Ejection fraction
EMB: Endomyocardial biopsy
HF: Heart failure
LGE: Late gadolinium enhancement
LV: Left ventricle
TTR: Transthyretin

## References

[1] Wechalekar A, Gillmore J, Hawkins P (2016). Systemic amyloidosis. Lancet.

[2] Rapezzi C, Lorenzini M, Longhi S (2015). Cardiac amyloidosis: the great pretender. Heart Fail Rev.

[3] Castellani C, Fedrigo M, Frigo AC, Della Barbera M, Thiene G, Valente M, Adami F, Angelini A (2017). Application of confocal laser scanning microscopy for the diagnosis of amyloidosis. Virchows Arch.

[4] Shore S, Grau-Sepulveda M, Bhatt D (2015). Characteristics, treatments, and outcomes of hospitalized heart failure patients stratified by etiologies of cardiomyopathy. JACC Heart Fail.

[5] Singh V, Tiemeier A, Malhotra S (2019). Coexistence of cardiac amyloidosis with coronary artery disease and the challenges in medical management. J Nucl Card.

[6] Neben-Wittich M, Wittich C, Mueller P (2005). Obstructive intramural coronary amyloidosis and myocardial ischemia are common in primary amyloidosis. Am J Med.

[7] Mueller P, Edwards W, Gertz M (2000). Symptomatic ischemic heart disease resulting from obstructive intramural coronary amyloidosis. Am J Med.

[8] Dorbala S, Cuddy S, Falk RH (2020). How to image cardiac amyloidosis: a practical approach. JACC Cardiovasc Imaging.

[9] Phelan D, Collier P, Thavendiranathan P (2012). Relative apical sparing of longitudinal strain using two-dimensional speckle-tracking echocardiography is both sensitive and specific for the diagnosis of cardiac amyloidosis. Heart.

[10] Musumeci M, Cappelli F, Russo D (2020). Low sensitivity of bone scintigraphy in detecting Phe64Leu mutation-related transthyretin cardiac amyloidosis. JACC Cardiovasc Imaging.

[11] Maurer M, Bokhari S, Damy T (2019). Expert consensus recommendations for the suspicion and diagnosis of transthyretin cardiac amyloidosis. Circ Heart Fail.

[12] Cheng Z, Cui Q, Tian Z (2013). Electron microscopy in patients with clinically suspected of cardiac amyloidosis who underwent endomyocardial biopsy and negative Congo red staining. Int J Card.

